# Difficult decisions in times of constraint: Criteria based Resource Allocation in the Vancouver Coastal Health Authority

**DOI:** 10.1186/1472-6963-11-169

**Published:** 2011-07-14

**Authors:** Craig Mitton, Francois Dionne, Rizwan Damji, Duncan Campbell, Stirling Bryan

**Affiliations:** 1 Centre for Clinical Epidemiology and Evaluation, Vancouver Coastal Health Research Institute, Vancouver, Canada; 2 School of Population and Public Health, University of British Columbia, Vancouver, Canada; 3 Vancouver Coastal Health, Vancouver, Canada

**Keywords:** priority setting, health care decision-making, disinvestment

## Abstract

**Objectives:**

The aim of the project was to develop a plan to address a forecasted deficit of approximately $4.65 million for fiscal year 2010/11 in the Vancouver Communities division of the Vancouver Coastal Health Authority. For disinvestment opportunities identified beyond the forecasted deficit, a commitment was made to consider options for resource re-allocation within the Vancouver Communities division.

**Methods:**

A standard approach to program budgeting and marginal analysis (PBMA) was taken with a priority setting working committee and a broader advisory panel. An experienced, non-vested internal project manager worked closely with the two-member external research team throughout the process. Face to face evaluation interviews were held with 10 decision makers immediately following the process.

**Results:**

The recommendations of the working committee included the implementation of 44 disinvestment initiatives with an annualized value of CAD $4.9 million, as well as consideration of possible investments if the realized savings match expectations. Overall, decision makers viewed the process favorably and the primary aim of addressing the deficit gap was met.

**Discussion:**

A key challenge was the tight timeline which likely lead to less evidence informed decision making then one would hope for. Despite this, decision makers felt that better decisions were made then had the process not been in place. In the end, this project adds value in finding that PBMA can be used to cover a deficit and minimize opportunity cost through systematic application of criteria whilst ensuring process fairness through focusing on communication, transparency and decision maker engagement.

## Background

As part of the fallout from the 2008 global economic crisis there has been extreme pressure on public sector spending. Across most countries, including Canada, deficits have increased dramatically leaving government departments having to either identify operational efficiencies or cut services. In the rare instance where additional investment in a particular area is made, such expansion is only achievable at a very real and potentially crippling effect on other departments, as was recently the case in the Province of Alberta. Interestingly, recent polls in Canada suggest that members of the public favor a reduction in public sector services over increases to income tax or sales tax. However, when specific cuts are identified, the cries of injustice are quick to make front-page news.

The fact is there are only so many resources to go around. This has been well documented in the health sector for many years and thus clearly pre-dates the most recent recession. What perhaps has made the recent move toward cuts in health care less palatable is that, in Canada as elsewhere, this has followed on the wake of over a decade of year on year real increases in expenditure. Nonetheless, the notion of decommissioning of health services has started to gain some traction. For example, the UK's National Institute of Health and Clinical Excellence (NICE) recently indicated a focus on appraisal of not only technologies for investment but also areas for disinvestment [1]. Several recent papers have also addressed the issue of disinvestment, discussing potential avenues for such action [2].

The Program Budgeting and Marginal Analysis (PBMA) framework was implemented in the Vancouver Coastal Health Authority (VCH) on a pilot basis from January to March 2010. VCH is one of six health authorities in British Columbia and with an annual operating budget of approximately CAD $3B provides services across the full continuum of care for about one million residents in Metro Vancouver. The specific focus of the exercise was within Vancouver Community Services, a major division within the health authority providing a mix of direct and contracted-out non-acute services. The primary aim of the project was to develop a plan to address the forecasted deficit of approximately $4.65 million for fiscal year 2010/11 on a $280 M budget. For disinvestment opportunities identified beyond the forecasted deficit, a commitment was made to consider options for resource re-allocation within the Vancouver Communities division.

This paper outlines the approach taken with respect to PBMA implementation and provides results that followed in terms of real decisions by the Senior Executive Team of the health authority. Lessons learned and areas for improvement are also described based on findings from a formal post-exercise evaluation with key decision makers. One challenge of PBMA identified in the past has been difficulty in generating and acting upon disinvestment options [3]. This action research project provides an example of success in terms of achieving disinvestments and thus factors for this are explored. In this, we consider the question as to whether PBMA can only have a large impact on disinvestment if there is external fiscal pressure. This paper should be acutely relevant to health care decision makers facing potential cuts of their own, as well as health services researchers likely to be called upon to support such activity.

## Methods

**Figure 1:**
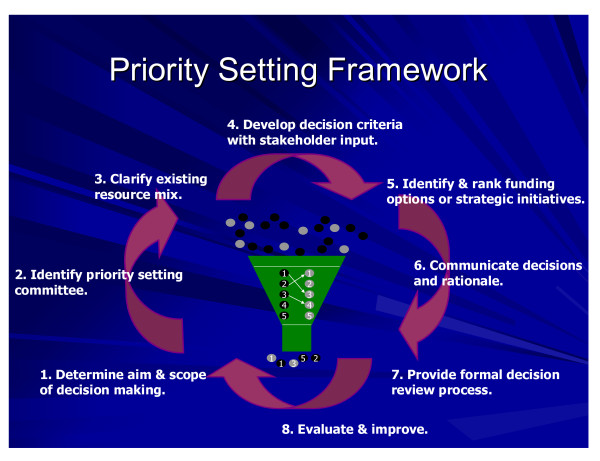
**PBMA approach as implemented in VCH**.

A standard approach to PBMA was taken [4], with a priority setting working committee comprised of all Directors and Clinical Leads from Vancouver Communities (n = 15), as well as a broader Advisory Panel that included a mix of Vancouver Communities personnel and Senior Executive members (n = 8). The Advisory Panel reported to the Senior Executive Team, which has final decision-making authority within the organization subject to Board approval as necessary. An experienced, non-vested internal project manager worked closely with the two-member external research team throughout the process. The research team acted as participant observers within an action research frame. The functions of each group are outlined in Table 1. The steps followed for PBMA implementation are depicted in Figure 1.

**Table 1:** Groups and roles

Groups	Roles
Priority setting working committee (Directors and Clinical Leads within Vancouver Communities division)	Actual implementation of PBMA, e.g., establishing criteria, deciding on process guidelines, rating of proposals, recommendations to Advisory Panel

PBMA Advisory Panel (mix of Vancouver Communities personnel and Senior Executive Team members)	Oversight of PBMA implementation, approval of the process guidelines, criteria and ratings, providing recommendations to the Senior Executive Team

Senior Executive Team (CEO and direct reports)	Review of the process recommendations and decisions on these recommendations (subject to Board approval)

Research team	Facilitators, process stewards, non-voting observers at working committee and advisory panel meetings

Operating on tight timelines (2.5 months total for process development, implementation and final decision making), process features included a formal communication plan, clearly defined, weighted assessment criteria linked to the strategic priorities of the health authority, a proposal rating tool and use of a standard business case template. The research team provided the working committee with a straw man set of criteria that were adapted and refined through several iterations. The criteria and rating tool are presented in Table 2. Criteria were weighted by a set of internal stakeholders through a simple point-allocation method. This involved asking approximately 20 managers and clinical leaders within VCH who were involved in the Vancouver Communities Division to allocate 100 points across the established criteria. A mean for each criteria was then calculated which was used as the weight in deriving the benefit score.

**Table 2:** Criteria and rating scale*

DOMAIN	*Domain Weights*	CRITERIA	DEFINITION	*Criteria Weight*	RATING SCALE						
					
					-3	-2	-1	0	1	2	3
		Alignment to Mandate	1) The service is directly related to health care or preventative health								
			2) The service is not provided by another organization outside VCH	*15*	Strong alignment with mandate (for releases)	Moderate alignment with mandate (for releases)	Weak alignment with mandate (for releases)	No alignment with mandate (for releases or investments)	Weak alignment with mandate (for invests)	Moderate alignment with mandate (for invests)	Strong alignment with mandate (for invests)
			3) The service is not the responsibility of an organization outside VCH								
		
Strategic alignment	*30*	Efficiency, Effectiveness and Appropriate-ness	1) Optimal use of resources to yield maximum benefits and results,								
			2) Care that is known to achieve intended outcomes	*5*	Goes against four or five of the five objectives	Goes against two or three of the five objectives	Goes against one of the five objectives	No net impact	Supports one of the five objectives	Supports two or three of the five objectives	Supports four or five of the five objectives
			3) Care provided is evidence based and specific to individual clinical needs								
			4) Promote wellness & prevention initiatives								
			5) Support clients at home/returning home or self management								
		
		Access	Impact on timely access to appropriate health care services for defined population(s). **Note**: the 'defined populations' are those using the services affected by the proposed changes.	*5*	Significant (more than 10%) worsening of the waiting times for more than 20% of the population or complete closure of a service	Significant (more than 10%) worsening of the waiting times 10% to 20% of the population or closure of a service for more than 25% of the time	Significant (more than 10%) worsening of the waiting times for less than 10% of the population or closure of a service for less than 25% of the time	No impact	Significant (more than 10%) improvement of the waiting times for less than 10% of the population or expansion of hours by less than 25%	Significant (more than 10%) improvement of the waiting times for 10% to 20% of the population or expansion of the hours by more than 25%	Significant (more than 10%) improvement of the waiting times for more than 20% of the population or opening of a new service facility (not a replacement)
		
		Flow/Integration	Impact on the coordination of health care services among programs to ensure flow and continuity of care from the patient's perspective (improve flow transitions)	*5*	Negative impact on continuity for more than 20% of clients or significant worsening for some patients	Negative impact on continuity for 10% to 20% of clients	Negative impact on continuity for 5% to 10% of clients	Impact on less than 5% of clients	Improvement in continuity for 5% to 10% of clients	Improvement in continuity for 10% to 20% of clients	Improvement in continuity for more than 20% of patients or significant improvement for some clients

Health Impact	*45*	Numbers affected	Number of individuals affected by the proposed change	*8*	More than 1500 have less services	251 to 1500 have less services	1 to 250 have less services	0	1 to 250 have more services	251 to 1500 have more services	More than 1500 have more services
		
		Equity	Impact on the health status of recognized groups where there is a known health status gap.	*10*	More than 50% of those affected are in the most disadvantaged groups (releases)	More than 50% of those affected are in moderately disadvantaged groups (releases)	10% to 50% of those affected are in the most disadvantaged groups (releases)	Less than 50% of those affected are in moderately disadvantaged groups (releases or investments)	10% to 50% of those affected are in the most disadvantaged groups (invests)	More than 50% of those affected are in moderately disadvantaged groups (invests)	More than 50% of those affected are in the most disadvantaged groups (investments)
		
		Significance of impact	Impact on clinical outcomes for the patient/client, including risk of adverse events, as compared to current practice/service.	*11*	Significant negative impact (either more than 25% of affected clients suffer negative effects or some clients are significantly affected)	Moderate negative impact (10% to 25% of affected clients suffer negative effects)	Small negative impact (5% to 10% of affected clients suffer some negative effects)	Less than 5% of affected clients experience any changes in satisfaction of safety	Small positive impact (5% to 10% of affected clients enjoy some positive effects)	Moderate positive impact (10% to 25% of affected clients enjoy positive effects)	Significant positive impact (either more than 25% of affected clients enjoy positive effects or some clients are significantly affected)
		
		Health promotion and disease prevention	Impact on illness and/or injury prevention, well-being and harm reduction as measured by projected longer term improvements in health	*8*	Significant negative impact on burden of disease	Moderate negative impact on burden of disease	Small negative impact on burden of disease	No impact on burden of disease	Small positive impact on burden of disease	Moderate positive impact on burden of disease	Significant positive impact on burden of disease
		
		Client experience	Impact on safety, effectiveness, and client experience of health service(s) provided.	*8*	Negative impact (safety or satisfaction) for more than 20% of affected clients	Negative impact (safety or satisfaction) for 10% to 20% of affected clients	Negative impact (safety or satisfaction) for 5% to 10% of affected clients	Less than 5% of affected clients experience any changes in satisfaction of safety	Positive impact (safety or satisfaction) for 5% to 10% of affected clients	Positive impact (safety or satisfaction) for 10% to 20% of affected clients	Positive impact (safety or satisfaction) for more than 20% of affected clients

		Workplace environment	Impact on workplace environment including morale, tools and equipment, personal and professional growth and teamwork	*5*	Significant negative impact on recruitment and retention or on stress leave	Moderate negative impact on recruitment and retention or on stress leave	Small negative impact on recruitment and retention or on stress leave	No impact	Small positive impact on recruitment and retention or on stress leave	Moderate positive impact on recruitment and retention or on stress leave	Significant positive impact on recruitment and retention or on stress leave
		
Organizational Impact	*25*	Innovation and knowledge transfer	Impact on the generation and/or application of new knowledge/practice.	*5*	Significant negative impact on the generation or application of new knowledge/practice	Moderate negative impact on the generation or application of new knowledge/practice	Small negative impact on the generation or application of new knowledge/practice	No impact	Small positive impact on the generation or application of new knowledge/practice	Moderate positive impact on the generation or application of new knowledge/practice	Significant positive impact on the generation or application of new knowledge/practice
		
		Implementation	Challenges to the implementation of proposed initiative (or reversal)	*5*	Significant political resistance and change would be very difficult to undo	Significant political resistance but change could be undone	Moderate political resistance expected	No political impact	Moderate political support expected	Strong political support expected	Strong political support and change can be reversed
		
		Downstream impact on service utilization	Impact of the proposed change on future use of health care services	*10*	Significant increase in future use of health care services	Moderate increase in future use of health care services	Small increase in future use of health care services	No impact on future use of services	Small decrease in future use of health care services	Moderate decrease in future use of health care services	Significant decrease in future use of health care services

*This table describes the overall domain, criteria, brief definition of criteria, weighting of criteria and rating scale for assessing each proposal.

Managers within Vancouver Communities submitted proposals for disinvestment and then investment to the working committee for assessment and ranking, with recommendations forwarded to the Advisory Panel. As a detailed program evaluation had previously been conducted, managers were intimately aware of the services across the program areas in this division. In some cases literature and other sources of information including benchmarking and practice patterns elsewhere were drawn on to develop proposals. In all cases, the type of evidence base for the given proposal was identified in the business case submission. Areas of care within the scope of the exercise are listed in Table 3, which constituted just under half of the total budget of the Vancouver Communities division. Issues related to scope are addressed below.

**Table 3:** Programs in scope of exercise

	**Direct Services In Scope**	**Contracted Services In Scope**	**Total In Scope**
		
			
		
Prevention and Promotion	18,964,284	50,296	19,014,580
		
			
		
Primary Care			
		
Primary Care	15,764,244	478,581	16,242,825
		
			
		
Adults and Older Adults	32,729,138	22,347,397	55,076,536
		
			
		
Alcohol and Drug	21,211,299	20,965,473	42,176,772
		
			
		
Mental Health	39,290,286	44,729,076	84,019,362
		
			
		
Special Care Contracts		2,153,626	2,153,626
		
			
		
Total Health Serv. and Supplies	127,959,251	90,724,449	218,683,700
		
			
		
Program Supports	18,173,063	0	18,173,063
		
			
		
Total Van. Comm. Hlth Services	146,132,314	90,724,449	236,856,763

The research team conducted an evaluation of the exercise immediately following announcement of final decisions by Senior Executive. The primary objective was to identify areas of improvement and assess possible expansion of PBMA to other areas within the health authority. Between March 23 and April 13, 2010, 10 decision-makers were interviewed face to face. Potential respondents were purposively selected by the research team to provide representation in terms of background (clinical and management) and level within the organization. The researchers held the identity of the respondents in confidence. The semi-structured interviews were audio taped and a thematic analysis was conducted. The results presented here focus on training, framework implementation, and future opportunities for PBMA within the health authority.

External ethics board approval was not sought for this project, as all of the people involved were health authority decision makers who were acting in the course of their normal duties. Further, the interviews to inform the evaluation were conducted solely to improve future implementation in the health authority.

## Results

The recommendations of the working committee included the implementation of 44 disinvestment initiatives with an annualized value of $4.9 million, as well as consideration of possible investments if the realized savings match expectations. These recommendations were approved by the Advisory Panel and then presented to Senior Executive on March 23, 2010. At that meeting, Senior Executive agreed to implement all of the process recommendations regarding disinvestments and to support further development of a limited set of investment options. For the latter, a preliminary list of investment options was established by the working committee for assessment of relative value against remaining disinvestment options that would not be used to meet the deficit. The working committee and Advisory Panel supported this conservative approach prior to receiving approval by the Senior Executive. The working committee in particular felt that expectations would be best held in check by not prematurely introducing a lengthy (and ultimately unrealistic) list of investment options.

The ranked disinvestment initiatives are presented in Table 4 (with actual program names removed), along with preliminary investment options in Table 5. Should further work on investments be pursued, the next step in this work would be to make relative value comparisons of each investment option to the next lowest ranked (i.e., least beneficial) disinvestment option, beyond that which was required to meet the deficit. If an investment option is deemed to be of greater value then the benefit lost by acting on the disinvestment option, a recommendation for resource re-allocation would ensue. Over time, such changes 'at the margin' would lead to improvements in the overall spending based on how well the objectives of the organization (as reflected through the criteria) are being met. As stated above, the primary aim of the exercise was to meet the deficit; changes beyond this were to be considered but were discretionary.

**Table 4:** Disinvestment options by weighted score

Disinvestment Opportunity	Weighted Score*	Annualized Savings**	VCH FTE impacted
1	0.44	$291,450	0.00

2	0.00	$76,690	-0.80

3	0.00	$42,686	-1.00

4	0.00	$182,439	0.00

5	0.00	$21,588	-0.50

6	0.00	$15,494	-0.40

7	0.00	$40,000	-0.60

8	0.00	$112,395	0.00

9	0.00	$17,741	-0.44

10	0.00	$48,400	-0.50

11	0.00	$50,700	-1.00

12	0.00	$57,886	-1.00

13	0.00	$77,000	-1.00

14	-0.25	$119,224	-1.00

15	-0.31	$48,215	-1.00

16	-0.31	$53,956	-0.70

17	-0.36	$120,000	-1.00

18	-0.50	$53,723	-0.60

19	-0.51	$42,000	-0.50

20	-0.53	$28,523	-0.16

21	-0.54	$100,906	-0.90

22	-0.56	$50,729	-0.10

23	-0.58	$96,498	-1.30

24	-0.58	$240,630	-2.80

25	-0.62	$762,715	-15.80

26	-0.65	$87,635	-1.00

27	-0.72	$64,340	-0.80

28	-0.73	$41,461	-0.50

29	-0.74	$41,897	-0.09

30	-0.76	$295,679	0.00

31	-0.83	$26,574	-0.30

32	-0.86	$60,000	0.00

33	-0.87	$88,776	0.00

34	-0.92	$21,000	0.00

35	-0.92	$70,605	-2.00

36	-0.96	$23,094	0.00

37	-1.00	$58,882	0.00

38	-1.04	$397,352	0.00

39	-1.04	$278,784	-4.00

40	-1.14	$120,000	0.00

41	-1.16	$68,000	0.00

42	-1.41	$134,500	-2.80

43	-1.45	$200,000	0.00

44	-1.64	$82,000	0.00

**TOTAL**		**$4,912,167**	**-44.59**

*A positive weighted score indicates that acting on the disinvestment would result in the organization moving closer to its objectives; zero weighted score indicates no impact on organizational objectives; a negative weighted score indicates that implementation would move the organization away from its objectives. **If all savings were realized as indicated, the deficit would be met by opportunity #42, leaving two proposals and approximately $282,000 for consideration against the investment opportunities.

**Table 5:** Investment options by weighted score

Investment Opportunity*	Weighted Score**	Annualized Investment***
1	2.07	$95,000

2	1.87	$69,600

3	1.72	$10,000

4	1.43	$45,000

5	0.44	$310,000

**TOTAL**		**$529,600**

*These proposals were put forward for consideration but were not acted upon immediately.

**Higher weighted scores indicate greater benefit gain by acting on the investment opportunity.

***If marginal analysis was to proceed beyond deficit elimination, re-allocation from disinvestment 43 to investments 1 to 3 would be recommended, as the benefit loss on the disinvestment is less then the potential benefit gained through these investments. Investment opportunity 4 and 5 would not proceed as the expected benefit gain is less then the benefit loss of disinvestment 44.

The results from the evaluation interviews are reported for three major themes: training, implementation and future use. Overall feedback on the training provided at the start of the project was positive. The main strengths of the training were that it was: focused (i.e. that it dealt with what was necessary to implement the PBMA process); direct (i.e. that it avoided jargon and was presented clearly and concisely); and relevant (i.e. it incorporated the use of examples that were meaningful to the participants). One concern that was raised with respect to training was that some participants had a misunderstanding early on about what specifically PBMA would do. That is, some of the participants expected PBMA to take the form of program evaluation whereby a ranking of all programs within Vancouver Communities would result. Additional time was required during training to explain that PBMA focuses on *changes to services *and the *marginal *or *incremental *benefit gain (or loss) from implementing a specific proposal. This has important ramifications because the focus of PBMA is on shifting the levels of service as opposed to complete deletion or novel introduction of a program (although in the extreme a shift in level could indeed be to fully eliminate or introduce a program). In moving forward, it was felt that two key aspects of the training should be emphasized: the use of real life examples in demonstrating the potential impact of PBMA and establishing that PBMA is principally about changes to services at the margin.

All respondents were extremely positive about framework implementation and resulting recommendations. There was recognition amongst respondents that the role of PBMA was primarily limited to the development of a plan to address the expected deficit. However, in that light, PBMA was viewed as being more robust then previous resource allocation processes that relied on historical patterns and/or politics. This was largely due to a well-defined set of decision criteria and high degree of process transparency. There was also clear recognition of the potential value of developing investment proposals and of considering re-allocations beyond what was required to balance the budget. Overall the results were seen as an improvement from previous activity for two main reasons: first, the marginal approach was seen to have led in many cases to a different set of proposals than what would have been otherwise considered; and second, the process enabled consideration of real changes to services instead of an ongoing search for greater efficiency. Open consideration of changes to services created a new range of alternative proposals to address the deficit problem.

A third major theme from the interviews related to future opportunities for the use of this priority setting process in the health authority. The clear message from the interviews was that PBMA is a desirable process and wider rollout should occur within VCH. This was based on three main points. First, the process provides a measurement methodology for the management of resources that was demonstrated to be effective in the Vancouver Communities pilot. Second, respondents felt that the process would lead to a more consistent approach to resource management across the entire organization. Third, because the process allows for marginal analysis across disparate services, the process creates opportunities for sharing of knowledge across the organization. It was felt that the rating process, specifically, would provide opportunities to exchange information between parts of the organization that do not tend to work together but whose decisions impact each other.

## Discussion

The logic of PBMA is straightforward: when resources are limited, decision makers must look at ways to re-allocate within the fixed pot available in order to receive the greatest return on investment. The PBMA process tends to bring a level of rigor and consistency to decision making seldom found within a historical and/or political allocation model [4]. The approach has been implemented in many organizations across countries [5], and where formal evaluation has taken place, decision makers almost universally indicate a desire to continue on with the process in future years subject to specific refinement [6]. Such support from the end-users is likely due at least in part to building in process characteristics such as transparency and use of best available evidence, both known to be key elements of fairness [7].

In the case of VCH, PBMA was implemented in a single division with a focus on primary care, community care and public health. To our knowledge few PBMA studies have been conducted in these areas. The primary aim of the exercise was to bring expenditure in line with available funds. The decision to use PBMA and to focus on Vancouver Communities as a pilot was the sole discretion of the Senior Executive of the health authority. As part of the PBMA process, decision makers must determine what activities are 'in scope' and what are 'out of scope'. In this case a little over half the Vancouver Communities budget was deemed to be out of scope for a variety of reasons including that some programs were ring-fenced, some programs received direct Ministry of Health funds and thus could not be challenged in this process, and in some cases programs were undergoing other separate reviews to address their deficit challenges. It was the working committee comprised of Vancouver Communities personnel that provided the recommendation for what was in and out of scope, and the deficit target was set accordingly. Some early discussion centered on the difficulties of making decisions in community services without acute services being represented at the table. Being explicit about this ensured that as proposals came forward, appropriate discussion with acute-based counterparts took place and generally it was felt that 'cost-shifts' from one part of the organization to another were avoided. Overall the process was viewed favorably by the end-users and the primary objective was met.

Nonetheless, one might anticipate at least three challenges to what was done with PBMA at VCH. First, as the timeline was very tight, proposal development may not have been as evidence based as one would hope. This was likely the case, although the application was at the level of managers and directors who have intimate knowledge of the programs under consideration. The leap was in drawing on evidence to support proposals for *changes *to existing services and as would be expected in some cases research evidence was available and in other cases 'softer' forms of evidence were relied upon. As reasonable evidence is a contributor to process fairness, one could argue that this process was not as fair as might be the case had longer timelines be in play and a stronger evidence base was sought. Ideally, managers and clinical leaders would receive support (either external from university-based researchers or internal from decision support personnel) to review the range of evidence for any given proposal in as thorough a manner as is possible given limited resources and time. Second, PBMA may not have lived up to its name because a formal re-allocation exercise did not immediately follow the allocation of agreed disinvestment proposals to the deficit. However, while the goal of PBMA is always to go deeper into the margin to elicit *options *for relative value assessment, it would not be correct to suggest that re-allocation did not occur in VCH. Disinvestment proposals were acted upon, with the released resources shifted to the corporate bottom line. That is, the opportunity cost of the disinvestments were assessed and it was felt that the benefit gain of these programs could be foregone in view of meeting the organization's budget requirements. In addition, all stakeholders expressed a desire to move forward with comparing the investment options to remaining (and perhaps additional) disinvestment options once the dust had settled. Third, it is not possible to know if the 'right' decisions were made with respect to disinvestment. Clearly the decision makers felt that *better *decisions were made then had the process not been used, but this is only a proxy of the true impact. In the absence of a real-world control, which would unlikely be plausible, one cannot be sure that what was done was right. Simply put, this is the reality of uncertainty in health care decision-making.

More generally, despite success across different settings, it is clear that the biggest stumbling block of PBMA is that which is at the very essence of choice making in health care: how to identify disinvestments alongside options for investment [3]. In this, PBMA stands no different then any other resource management tool. In order for 'success' to be achieved, decision makers must be 'bought in' to acceptance of scarcity and the need to assess options for change, there must be strong leadership, and a high level of trust must exist between managers and clinicians. These are but a few key organizational attributes found in the literature that can help envisage the outcome of a given priority setting exercise. But is that enough? Is it the case that an external impetus such as a projected deficit is required in order to achieve disinvestment targets?

For the exercise reported herein, the primary aim was to implement a more rigorous, criteria-based process to achieve a disinvestment target based on a projected deficit for the Vancouver Communities division. Thus success in this project can be measured against nothing less then whether this was achieved. That the working group went further to produce more disinvestment options then the deficit called for perhaps suggests that the decision makers had bought in to the principle of PBMA as a re-allocation tool. Those interviewed suggested that approaching programs for disinvestment at the margin resulted in a different set of options then would have resulted through standard program evaluation (and undoubtedly different then across the board percentage cuts). So while there was pain in the cuts, it was collectively held that what was put forward was the least painful set of options (as measured against the objectives of the organization which were reflected in the criteria). At the same time, there can be no denying that the external fiscal impetus 'forced their hand'. It seems that the decision makers got to a different place then they would have had they not implemented PBMA but it is not clear that the decision makers would have engaged in a new method of priority setting had there not been an external impetus. In VCH, deficits had been projected in previous years but the difference this year, it would seem, was a clear government directive of no bailouts. So strictly speaking it was not just the external impetus of a fiscal constraint but the external political will to not 'allow' deficits to stand, which was very different then previous years.

Importantly, regardless of the fiscal driver, the 'selling point' of PBMA must remain as a process that can guide decision making towards optimal allocation of limited resources. Put another way, proactive resource management requires continual assessment of existing services vis-à-vis new investment options and where the relative value of the latter outweighs the former resources should be shifted accordingly [8]. This is the case whether there is a deficit, a surplus, or the organization is in a neutral budget state. This is also the case whether PBMA is being considered or some other framework or process is taken up to assist decision makers in resource allocation. Thus in response to the above question, based on our own experience and what is reported in the literature, our view is that it is not necessary to have an external fiscal driver in play but at the same time it can serve as a very helpful lever.

One question for VCH is how to proceed so that the success within Vancouver Communities can be built upon in other areas while also mitigating potential challenges. In our view, major gains in terms of shifting resources to better meet organizational objectives is best achieved through systematic application of the framework across divisions. In that, several points should be considered. First, the willingness of decision-makers to participate depends on their trust in the process. If the only perceived outcome is a likely loss of resources, decision-makers may be reticent to participate and no process can overcome a 'forced participation'. This relates to what Jan calls 'credible commitment' [9]. Second, as the use of PBMA widens, more disparate services will be included in the process (eventually including a mix of acute care and population health services). As others have found, this is the real *raison d'etre *of multi-criteria decision analysis as it provides a method of assessment that relates to the many different (and at times competing) objectives of the decision maker [10]. Finally, there is always a risk of over reliance on the actual rating score for each proposal. The assessments that lead to these ratings, and the underlying assumptions, should always be critically analyzed. As was done in the exercise described herein, the quantitative assessment can serve as the basis for consensus decision-making.

A final thought for further rollout within VCH is to highlight the importance of the sequential buy-in that took place in the Vancouver Communities exercise. Because the training was viewed favorably there was early buy-in for the process. Then, because of broad participation in criteria development, there was buy-in for the notion of how 'benefit' would be defined. Next, there was buy-in into the marginal approach to proposal development and assessment. And finally, building on the previous steps, there was buy-in into the rating of proposals as well as the final rankings. In the end, this led to a plan that had consensus approval of the working committee. Engagement by the Advisory Panel early on and throughout the process ensured that there were no surprises at that level. In addition, the Advisory Panel took its role seriously and was able to provide a strategic lens that complemented the operational lens of the working committee. In short, buy-in and building from within can be seen as key strategies for further use of PBMA in VCH and elsewhere.

## Conclusion

In the end, the potential of moving to an organization-wide application of PBMA in VCH will hinge most directly on the broader organizational context (e.g., does it have a 'learning culture'), as well as executive endorsement and clinical leadership. In this, if the focus is simply to meet a projected deficit based on explicit criteria, the PBMA process is being sold well short. That PBMA can respond should this be the objective was clearly demonstrated in the Vancouver Communities exercise. Case studies both in Canada and elsewhere have demonstrated success in terms of identifying disinvestment options sufficient to meet a budget gap and also enable assessment for resource re-allocation across services. Some stakeholders may view this through the lens of 'winners' and 'losers' but the process should focus on the *net impact*, in terms of ability to meet system objectives, for the *entire *population being served. It is with this in mind, regardless of the fiscal reality of the day, that will place decision makers in a position to take up a framework like PBMA to improve resource management activities.

## Competing interests

The authors declare that there are no competing interests.

## Authors' contributions

CM and FD drafted the paper and participated in study design. RD, DC and SB participated in study design and provided detailed comments on the paper. The authors read and approved the final manuscript.

## Pre-publication history

The pre-publication history for this paper can be accessed here:

http://www.biomedcentral.com/1472-6963/11/169/prepub
